# Influence Mechanism of Silicon on Carbide Phase Precipitation of a Corrosion Resistance Nickel Based Superalloy

**DOI:** 10.3390/ma13040959

**Published:** 2020-02-21

**Authors:** Tao Liu, Mei Yang, Fenfen Han, Jiasheng Dong

**Affiliations:** 1 School of Materials Engineering, Jiangsu University of Technology, Changzhou 213001, China; 2 Shanghai Institute of Applied Physics, Chinese Academy of Sciences, Shanghai 201800, China; 3 Institute of Metal Research, Chinese Academy of Sciences, Shenyang 110016, China

**Keywords:** Ni-Mo-Cr based superalloy, silicon, diffusion, carbide, precipitation

## Abstract

The effect of silicon on diffusion behavior of the carbide forming elements in Ni-Mo-Cr-Fe based corrosion-resistant alloy is studied by diffusion couple experiment. One group of diffusion couples are made of the alloy with a different silicon content, another group of diffusion couples are made of pure nickel and the alloy with different silicon content (0Si, 2Si). Two groups of alloys with same silicon content and different carbon content are also prepared, the microstructure of solution and aging state of these two groups alloys are analyzed, and their stress rupture properties are tested. The effect of silicon on the diffusion of alloy elements and the interaction effect of carbon and silicon on the microstructure and stress rupture properties of the alloy are analyzed. The mechanism of Si on the precipitation behavior of carbide phase in Ni-Mo-Cr-Fe corrosion resistant alloy is discussed. The results show that silicon can promote the diffusion of carbide forming elements and the formation of carbide. The precipitation behavior of the secondary phase is the result of the interaction effect of silicon and carbon, and is related to the thermal history of the alloy. Combined with the characteristic of primary carbides, it is confirmed that the precipitation of M_12_C type secondary carbide is caused by the relative lack of carbon element and the relative enrichment of carbide forming elements such as molybdenum. The stress rupture properties of two silicon-containing alloys with different carbon contents in solution and aging state are tested. The stress rupture life of low carbon alloy is lower compared with high carbon alloy at solution state, but after aging treatment, the stress rupture life of low carbon alloy is significantly improved, and higher than that of high carbon alloy. The main aim of this research is to reveal the influence mechanism of silicon on carbide phase precipitation of a Ni-Mo-Cr-Fe based corrosion-resistant superalloy, which provides theoretical basis and reference for later alloy design and engineering application.

## 1. Introduction

Ni-Mo-Cr-Fe based superalloy has excellent corrosion resistance and high temperature resistance, which is used as a structural material in nuclear power molten salt breeder reactor environments [1,2]. The structural parts used in molten salt breeder reactors require long-term service, generally more than 30 years. Consequently, the alloy needs stable microstructure and reliable performance, otherwise it will create potential safety hazards with serious consequences [3].

The alloy has the problem of corrosion resistance, irradiation resistance, and oxidation resistance, and these properties can be improved by adding Si [4,5,6]. The existing research results show that Si will affect the high temperature brittleness of the alloy and affect the welding and high temperature condition properties [7,8]. Silicon was also reported to have great effect on formation of carbides [1,2,9,10,11], and the silicon element can stabilize and improve the high temperature properties of the alloy [12,13,14]. Liu et al. conducted a preliminary study on Ni-Mo-Cr-Fe base alloy, and found that the addition of Si will affect the secondary phase precipitation type of high temperature aging and low temperature long-term aging of the alloy, the precipitation phase of silicon-free alloy is M_6_C type, while that of silicon containing alloy is M_12_C type carbide phase [1]. Because the M_12_C phase has a coherent orientation with the matrix, it can significantly increase the high-temperature endurance strength of the alloy, while the M_6_C secondary phase has no coherent orientation with the matrix, so the strengthening effect is not as good as M_12_C phase [2].

Large amounts of research papers have reported that the distribution, size, amount, morphology, and type of carbide phase all have a great influence on the properties of the alloy [15,16,17,18]. Some researchers have found that the element concentration, element type, element ratio, heat treatment temperature, time, and other factors all have influence on the precipitation of the carbide phase [19,20,21,22,23], however, in essence, carbide is formed by element diffusion, while carbide formation needs the required metal element and carbon element reach the necessary element concentration for carbide formation, which rely on element diffusion [24,25,26,27,28,29]. Some researchers have shown that Si has an effect on the precipitation rate of alloy and can delay the transformation from ε/ε’-carbides to cementite [30,31]. The Ni-Mo-Cr-Fe based superalloy used for molten salt breeder reactors was strengthened by solid solution of molybdenum and Cr, and the dispersed primary and secondary carbides, the carbides have great influence on the properties of the alloy [32,33]. However, at present, there is no research reporting on the mechanism of Si affecting carbide precipitation, and it is not clear how Si affects the secondary phase precipitation of the alloy. Whether the secondary phase type of the alloy are caused by the influence of Si on element diffusion. It is of great significance to understand the mechanism of different secondary precipitation types for the safe use of the alloy, for the optimization of the properties of the alloy and even for the optimization of the properties of other Si containing alloy materials. High temperature and high pressure exist in the service environment, which may lead to creep damage. The carbide phase in this kind of alloy is an important strengthening means, and the secondary phase has a great influence on the properties of the alloy. In this paper, the influence mechanism of Si on primary and secondary phase precipitation is studied, the results of this study reveal the precipitation mechanism of silicon and carbon on the carbide phase in the alloy, and provide theoretical support for further optimization and engineering application of the alloy.

## 2. Materials and Methods

The chemical composition (wt.%) of the experimental alloy is given in Table 1. The master alloy was melted into 10 kg ingot via vacuum induction melting (VIM) furnace. The ingot was homogenized and subsequently forged into bars of Φ30 mm in cross section. 

Samples were cut from these round bars using an electric discharge machine (EDM). Three types of diffusion couples (0.09Si-2Si, 0Si-pure nickel, and 2Si-pure nickel) were used to test the influence of Si on the diffusion behavior of alloy elements. The preparation method of diffusion couple is to cut a sample bar with a length of 10 mm by using EDM, and the two sides of the sample bar are required to have high parallelism. Metallographic grinding were carried out on the sample, both sides shall be sanded to 5000 meshes in turn, and then polished. The two surfaces with better polishing shall be cleaned with alcohol, and absence of scratches is required under the microscope. The diffusion couple is connected with stainless steel plate, the fixture material of diffusion couple is made of high temperature resistance stainless steel. When two samples with different components are butt jointed, reliable physical contact shall be realized to ensure the close combination of diffusion interface and the effect of diffusion experiment. By applying pressure to the sample group of diffusion couple with bolts, the steel plates on both sides shall reach a certain plastic deformation to increase the connection pressure, so as to ensure the close combination of diffusion interface and the diffusion experiment effect. The heating equipment of diffusion couple is KBF/1400 °C box type resistance furnace. The diffusion samples were heated with the furnace, and the diffusion temperatures were 650, 800, and 1000 °C, respectively, and the holding time was 200 h, and then air-cooled. All the diffusion samples have no pre-heat treatment, just forged state alloy.

The stress rupture tests were conducted at 650 °C/325 MPa in an electronic high temperature creep endurance tester, in air. The solid solution heat treatment regime of alloy is 1180 °C/1 h, and aging heat treatment regime of the alloy is 1180 °C/1 h + 900 °C/2 h, water quenching. Samples for optical microscopy (OM), and scanning electron microscopy (SEM, HITACHIS-3400N, HITACHI, Japan) observation were mechanically polished. The samples were etched by a solution of 3 g CuSO_4_ + 10 mL H_2_SO_4_ + 40 mL HCl + 50 mL H_2_O. The electronic probe microanalysis (EPMA, EPMA-1610, Shimadzu, Japan) was employed for compositional analysis and phase identification. The SEM with combination of energy dispersive spectrum (EDS, Oxford X-max 80, Oxford, UK) was used in the present experiment.

## 3. Results

### 3.1. Microstructure and Element Distribution after Diffusion

#### 3.1.1. Diffusion Couple of 0.09Si-0.45Si Alloy

(a) 650 °C/200 h diffusion

**Figure 1:**
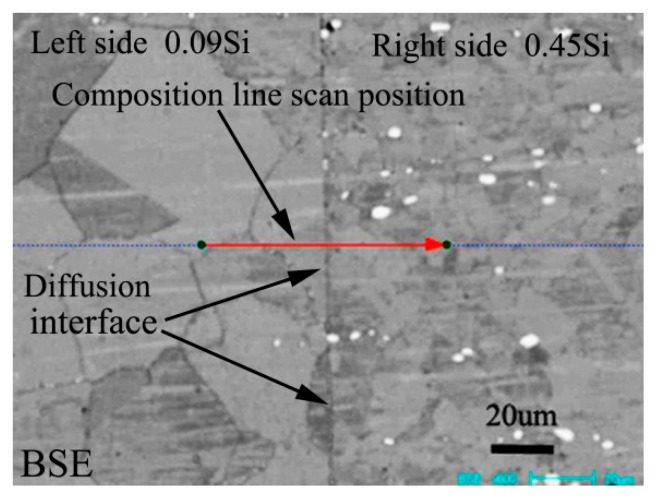
Microstructure of diffusion couple sample after 650 °C/200 h heat treatment: left side is 0.09Si alloy and right side is 0.45Si alloy.

Figure 1 shows the BSE morphology of diffusion couple composed of 0.09Si alloy and 0.45Si alloy after diffusion at 650 °C for 200 h. The left side of the figure is the microstructure of 0.09 Si alloy and the right side is the 0.45Si alloy. The red line shown in the figure is the position and direction of component line scanning by EPMA.

**Figure 2:**
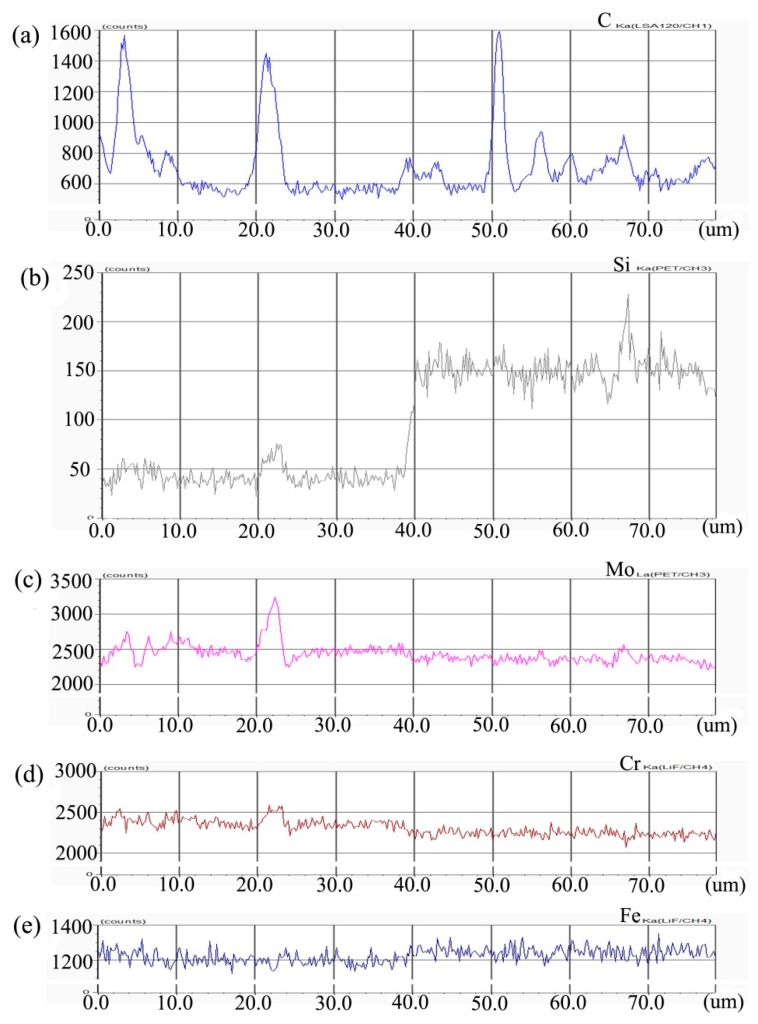
Composition distribution curve of the diffusion couple sample of 0.09Si and 0.45Si alloy after 650 °C/200 h heat treatment: (**a**) C, (**b**) Si, (**c**) Mo, (**d**) Cr, and (**e**) Fe. (The horizontal coordinate of the figures is the scanning distance and the vertical coordinate is element signal intensity, the zero position is the starting point of scanning).

Figure 2 shows the composition distribution curve of EPMA analysis in diffusion couple after diffusion of Mo, Cr, Fe, C, and Si. The bonding position of diffusion couple interface is at the position of 40 μm. The left side of the diffusion couple is 0.09Si alloy and the right side is 0.45Si alloy. From the diffusion curve of Si, it can be seen that Si diffuses from high concentration area to low concentration area, resulting in a diffusion range of nearly 2 μm. The diffusion curve of other elements shows that the content of these elements in the two alloys is similar, and the curve height on both sides is similar. During the diffusion experiment, these elements did not show the phenomenon of upward diffusion.

(b) 1000 °C/200 h diffusion

**Figure 3:**
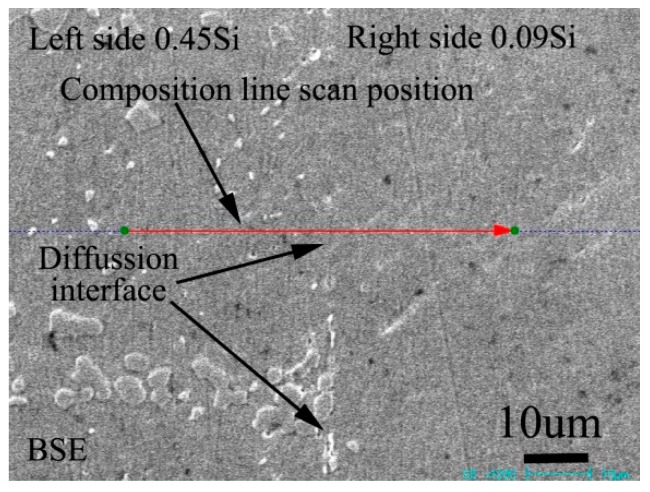
Microstructure of diffusion couple sample after 1000 °C/200 h heat treatment: left side is 0.45Si alloy and right side is 0.09Si alloy.

Figure 3 shows the BSE morphology of the diffusion couple composed of 0.45Si alloy and 0.09 Si alloy after 1000 ℃/200 h diffusion. The left side of the figure is the microstructure of the alloy with 0.45Si and the right side is the microstructure of the alloy with 0.09 Si. The red line in the figure shows the position and direction of EPMA line scanning.

**Figure 4:**
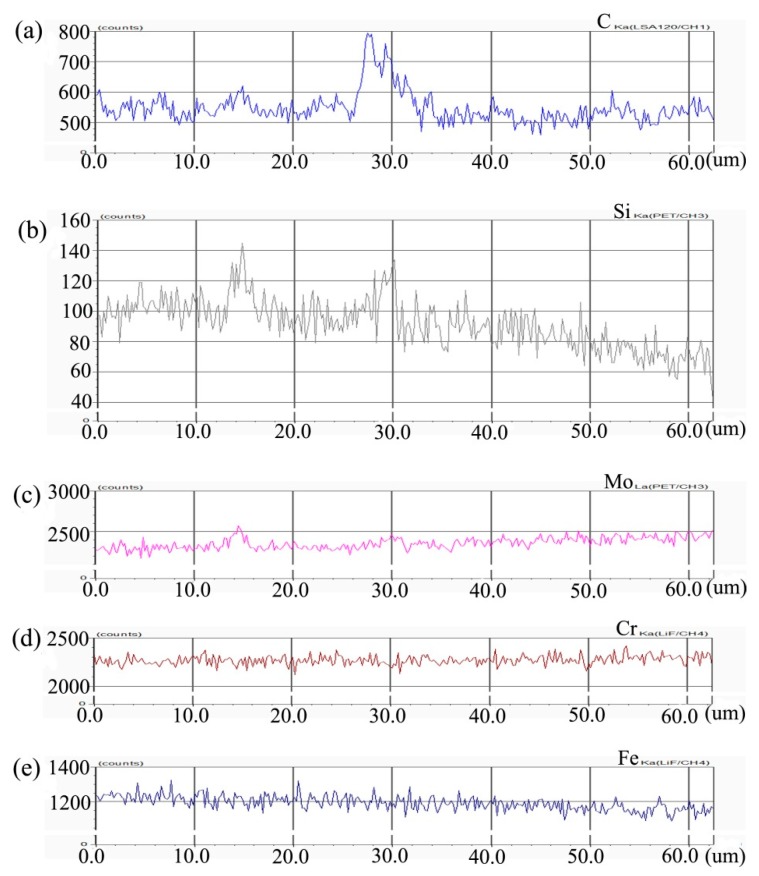
Composition distribution curve of the diffusion couple sample of 0.45Si and 0.09Si alloy after 1000 °C/200 h heat treatment: (**a**) C, (**b**) Si, (**c**) Mo, (**d**) Cr, and (**e**) Fe. (The horizontal coordinate of the figures is the scanning distance and the vertical coordinate is element signal intensity, the zero position is the starting point of scanning).

Figure 4 shows the distribution curve of Mo, Cr, Fe, C, and Si in the diffusion couple after diffusion. The interface of diffusion couple is located at 31.1 μm, the left side of the figure is the alloy with 0.45Si and the right side is the alloy with 0.09 Si. From the diffusion curve of Si, we can see that Si diffuses from high concentration area to low concentration area, resulting in a diffusion range of nearly 30 μm. The Si diffusion range at 1000 °C is much larger than that of 650 °C. Because of the contents of Mo, Cr, and Fe in the two alloys are similar, the diffusion curves of these elements show that the curves on both sides are similar. During the diffusion experiment, these elements did not show the phenomenon of upward diffusion.

#### 3.1.2. Diffusion of Pure Nickel-Alloy Couple at 800 °C for 200 h

(a) 800 °C/200 h diffusion of pure nickel-0Si alloy

**Figure 5:**
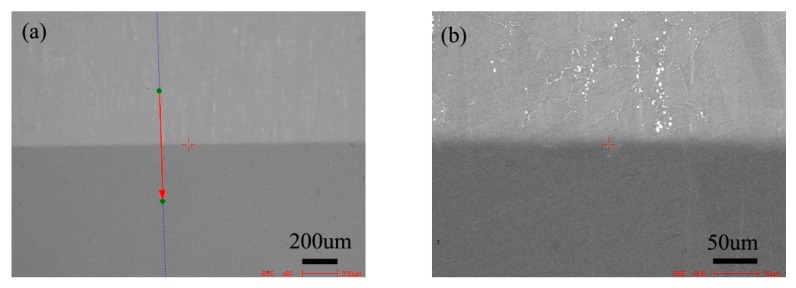
Microstructure of diffusion couple sample after 800 °C/200 h heat treatment: (**a**) composition line scan position of the sample and the upper side is 0Si alloy and lower side is pure nickel, (**b**) the enlarged view of figure a.

Figure 5 is the BSE morphology of the diffusion couple composed of the 0Si alloy and pure nickel after diffusion at 800 °C for 200 h. The upper side of the figure is the microstructure of silicon free alloy and the lower side is the microstructure of pure nickel. The first map is the location map of line scanning, and the second is the enlarged map.

**Figure 6:**
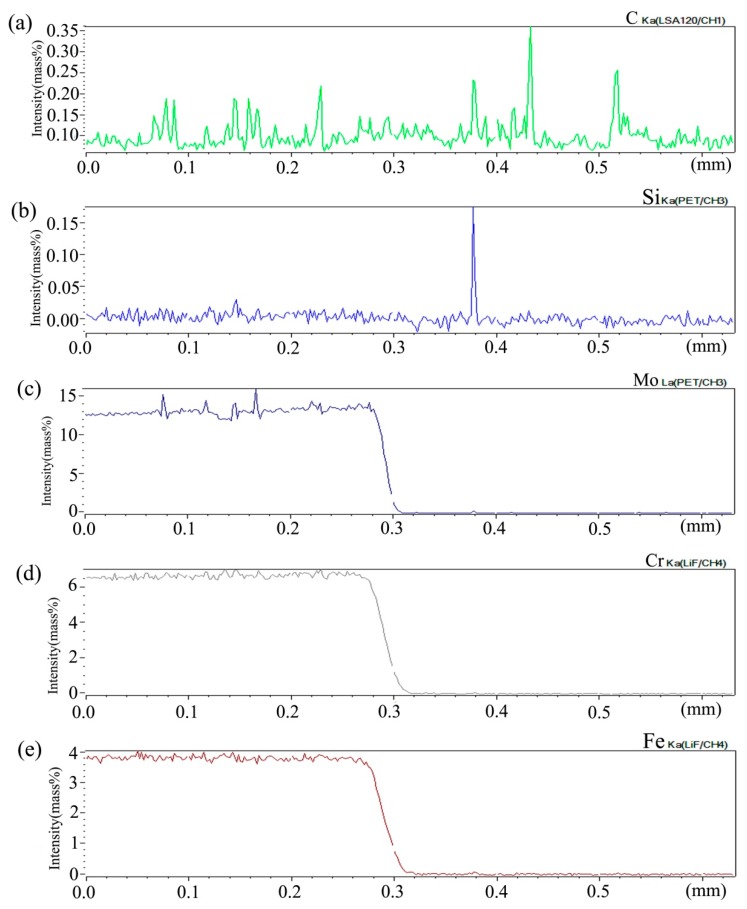
Composition distribution curve of the diffusion couple sample of 0Si alloy and pure nickel after 800 °C/200 h heat treatment: (**a**) C, (**b**) Si, (**c**) Mo, (**d**) Cr, and (**e**) Fe. (The horizontal coordinate of the figures is the scanning distance and the vertical coordinate is element signal intensity, the zero position is the starting point of scanning).

Figure 6 shows the distribution curve of Mo, Cr, Fe, C, and Si in the diffusion couple after diffusion. The bonding position of diffusion couple interface is 0.315 mm, the left side is silicon free alloy, and the right side is pure nickel. It can be seen from the diffusion curve of Si that the curve is almost straight without Si, and there is no significant concentration gradient change. From the diffusion curve of C element, it can be seen that the curve on both sides is almost equal in height, there is no significant concentration gradient change, and the diffusion speed is very fast. In the measurement range, the distribution is almost uniform. The diffusion curve of other elements shows that the diffusion speed is relatively slow and a significant concentration gradient can be seen. From the diffusion range, the range of Cr is larger than that of Fe, and the range of Mo is the smallest one, which also shows that the diffusion rate of multi-element decreases one by one, and Mo is the slowest. The diffusion range of the Si is 0.061 mm, and Cr is 0.058 mm, Fe is 0.057 mm, and Mo is 0.035 mm.

**Figure 7:**
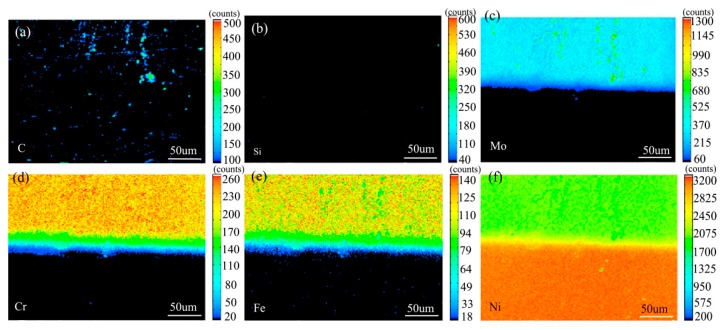
Composition distribution map of the diffusion couple sample of 0Si alloy and pure nickel after 800 °C/200 h heat treatment: (**a**) C, (**b**) Si, (**c**) Mo, (**d**) Cr, (**e**) Fe, and (**f**) Ni.

Figure 7 shows the element distribution map of the diffusion couple sample of 0Si alloy and pure nickel after 800 °C/200 h heat treatment by EPMA. It can be seen from the element distribution map of C that carbon element is distributed in both alloy and pure nickel. Si distribution map shows that there is no Si in alloy and pure nickel. The diffusion range of Cr and Fe is obviously wider than that of Mo. In addition, from the diffusion range of Cr and Fe, after the elements near the interface diffuse to pure nickel, the elements in the matrix can quickly supplement to the dilution layer, so there are diffusion layers with different concentration levels (color representation). However, when Mo diffuses into pure nickel at the interface, the diffusion ability of elements in the matrix to the Mo dilution layer is limited, so there is no diffusion image like Cr and Fe. The analysis and comparison of Mo map show that a large amount of secondary phases precipitated along the grain boundary and enriched a large amount of Mo, while the silicon free alloy did not have a large amount of enrichment. The presence of Si promotes the diffusion of Mo, Fe, and Cr in the secondary phase precipitation temperature, promotes the secondary phase precipitation, mainly the Mo rich secondary phase precipitation.

(b) 800 °C/200 h diffusion of pure nickel-2Si alloy

**Figure 8:**
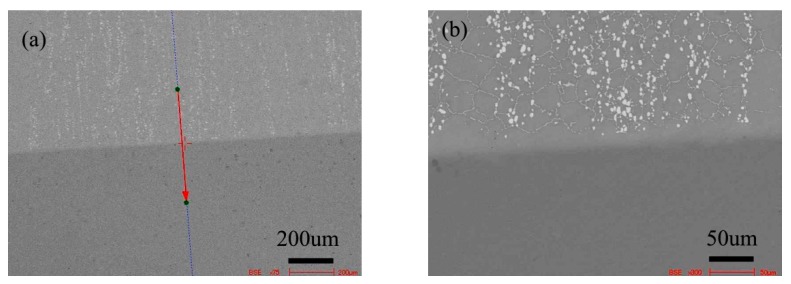
Microstructure of diffusion couple sample after 800 °C/200 h heat treatment: (**a**) composition line scan position of the sample and the upper side is 2Si alloy and lower side is pure nickel material, (**b**) the enlarged view of figure a.

Figure 8 shows the microstructure of the diffusion couple of the 2Si alloy and pure nickel after 800 °C/200 h diffusion. The upper side of the figure is the microstructure of 2Si alloy and the lower side is the microstructure of pure nickel. The first figure is the location map of line scanning, and the second is the enlarged map.

**Figure 9:**
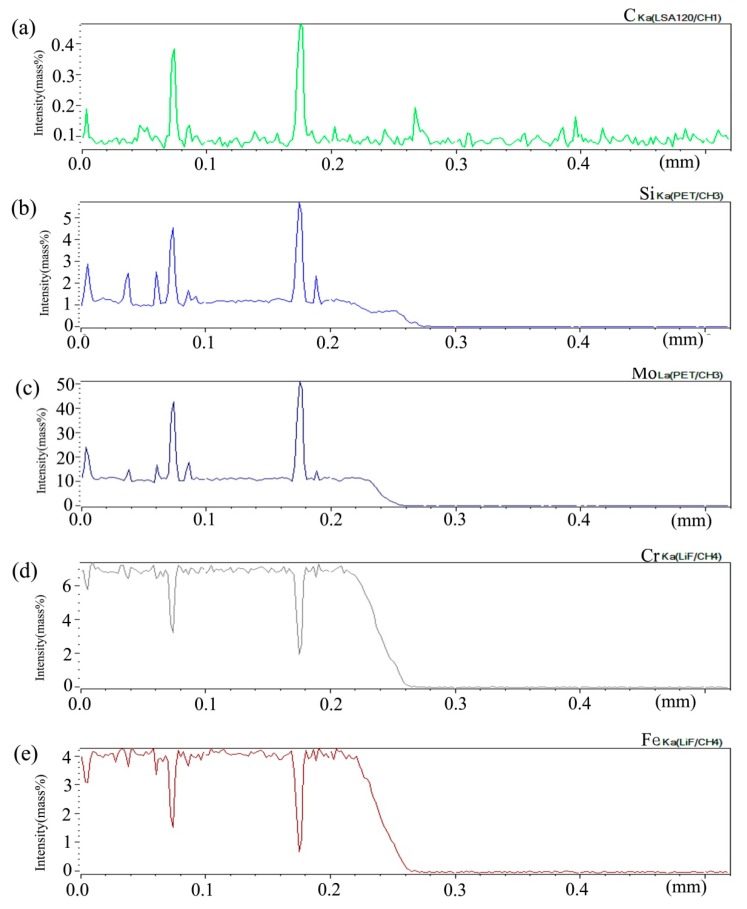
Composition distribution curve of the diffusion couple sample of 2Si alloy and pure nickel after 800 °C/200 h heat treatment: (**a**) C, (**b**) Si, (**c**) Mo, (**d**) Cr, and (**e**) Fe. (The horizontal coordinate of the figures is the scanning distance and the vertical coordinate is element signal intensity, the zero position is the starting point of scanning).

Figure 9 shows the distribution curve of Mo, Cr, Fe, C, and Si in the diffusion couple after diffusion. The bonding position of diffusion couple interface is at the position of 0.26 mm, the left side is silicon free alloy, and the right side is pure nickel. The curve in the figure is the element distribution curve of C, Si, Mo, Cr, and Fe. From the diffusion curve of carbon element, it can be seen that the curve on both sides is almost equal in height, there is no significant concentration gradient change, and the diffusion speed is very fast. In the measurement range, the distribution is almost uniform.

From the diffusion curve of Si, it can be seen that the diffusion of Si is significant, the diffusion distance is 0.078 mm, and the dilution range of Si appears on the left side of the interface. The diffusion curve of other elements shows that the diffusion speed is relatively slow and a significant concentration gradient can be seen. From the diffusion range, the diffusion range of Cr is 0.062 mm which is larger than that of Fe and the diffusion range of Fe is 0.060 mm which is larger than that of Mo which has the diffusion range 0.043 mm, this also shows that the diffusion rate of multi-element decreases one by one, and Mo is the slowest. Compared with the concentration gradient curves on the left and right sides of the interface, it is found that the dilution gradient of Si is smaller, which indicates that the Si from the matrix is added to the dilution at a faster speed, and that the diffusion speed of Si is much higher than that of Cr, Fe, and Mo.

**Figure 10:**
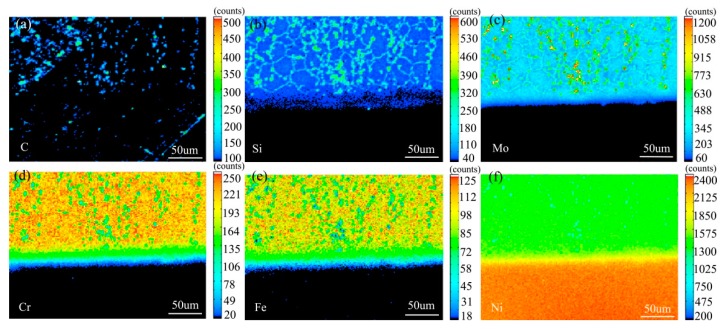
Composition distribution map of the diffusion couple sample of 2Si alloy and pure nickel after 800 °C/200 h heat treatment: (**a**) C, (**b**) Si, (**c**) Mo, (**d**) Cr, (**e**) Fe, and (**f**) Ni.

Figure 10 shows the element surface distribution map of the diffusion couple sample of 2Si alloy and pure nickel after 800 °C/200 h heat treatment by EPMA. It can be seen from the element distribution map of C that carbon elements are distributed in alloy and pure nickel, and the diffusion distance is far, and the diffusion speed is the fastest. Si distribution map shows that Si in alloy diffuses into pure nickel. Cr, Fe, and Mo also diffused into pure nickel. It is clear from comparison of the distribution map of these elements that the diffusion range of Si is far greater than that of Cr and Fe, while the diffusion range of Mo is the smallest. In addition, from the diffusion range of Cr and Fe, after the elements near the interface diffuse to pure nickel, the elements in the matrix can quickly supplement to the dilution layer, so there are diffusion layers with different concentration levels (color representation). However, when Mo diffuses into pure nickel at the interface, the diffusion ability of elements in the matrix to Mo dilution layer is limited, so the character of Mo diffusion map is not like that of Cr and Fe. The diffusion map of Si shows that the concentration of Si in nickel is very close to the color of the matrix, indicating that Si diffusion speed is faster.

The analysis and comparison of Mo map show that a large amount of secondary phases precipitated along the grain boundary which enriched Mo, while the silicon free alloy did not have Mo enrichment. The presence of Si in the secondary phase precipitation temperature promotes the diffusion of Mo, Fe, and Cr elements, promotes the secondary phase precipitation, mainly the Mo rich secondary phase precipitation.

#### 3.1.3. Diffusion of Pure Nickel-Alloy Couple at 1000 °C for 200 h

(a) 1000 °C/200 h diffusion of pure nickel-0Si alloy

**Figure 11:**
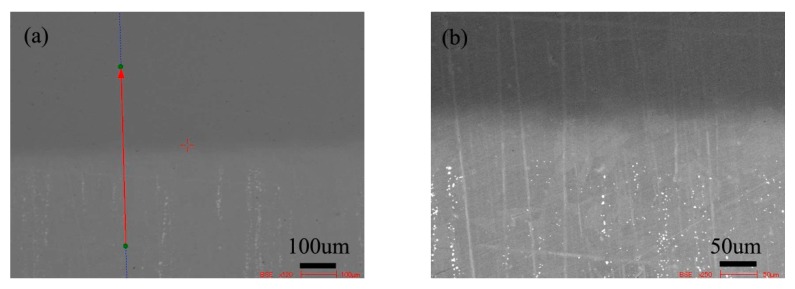
Microstructure of diffusion couple sample after 1000 °C/200 h heat treatment: (**a**) composition line scan position of the sample and the upper side is pure nickel material and lower side is 0Si alloy, (**b**) the enlarged view of map a.

Figure 11 shows the microstructure of diffusion couple of 0Si and pure nickel after diffusion at 1000 °C/200 h. The lower side of the map is the microstructure of silicon free alloy and the upper side is the microstructure of pure nickel. The first map is the location map of line scanning, and the second is the enlarged map of the first map.

**Figure 12:**
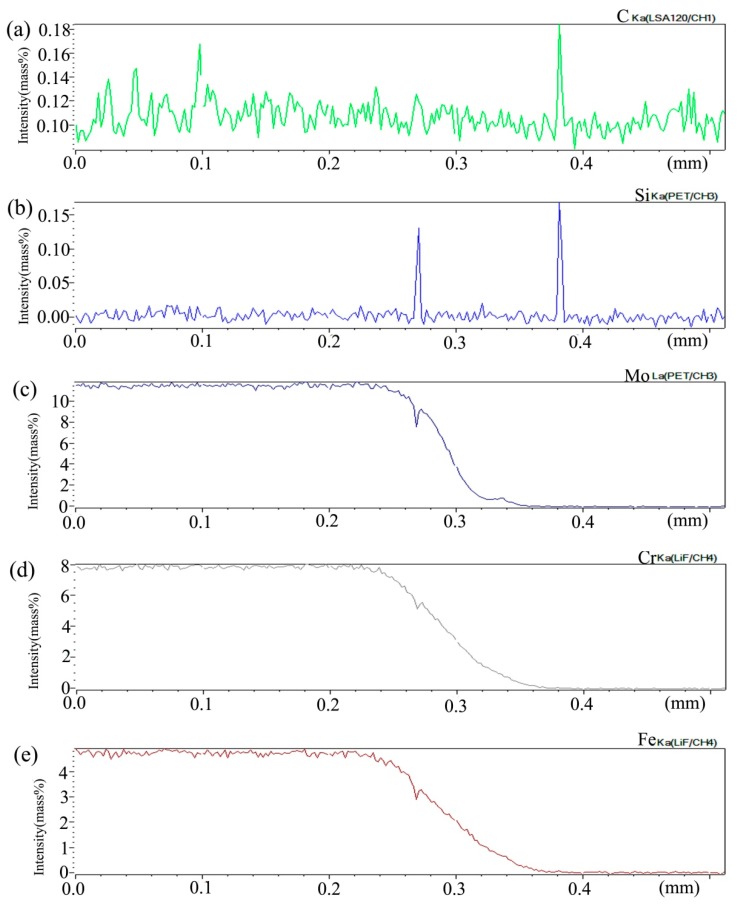
Composition distribution curve of the diffusion couple sample of 0Si alloy and pure nickel after 1000 °C/200 h heat treatment: (**a**) C, (**b**) Si, (**c**) Mo, (**d**) Cr, and (**e**) Fe. (The horizontal coordinate of the figures is the scanning distance and the vertical coordinate is element signal intensity, the zero position is the starting point of scanning).

Figure 12 shows the distribution curve of Mo, Cr, Fe, C, and Si in the diffusion couple after diffusion. The bonding position of diffusion couple interface is at 0.255 mm position. The left side of the figure is silicon free alloy, and the right side is pure nickel. From the diffusion curve of C element, it can be seen that the curve on both sides is almost the same height, there is no significant concentration gradient change, and the diffusion speed is very fast. In the measurement range, the diffusion distance is far. It can be seen from the diffusion curve of the Si that the curve is almost straight because the alloy did not have Si, and there is no significant concentration gradient change. The diffusion curve of other elements shows that compared with the low temperature diffusion, the diffusion speed of all elements is faster and the diffusion distance is longer when the temperature is increased and the diffusion time is the same. There is still a significant concentration gradient.

From the curves trend on the left and right sides of the interface, compared with that of the low-temperature diffusion curves, it can be seen clear that under the high-temperature diffusion condition, the highest component curve of each element on one side of the alloy moves towards the interface, which shows that after the element near the interface diffuses to pure nickel at high temperature, the element in the matrix can diffuse to the interface at a faster speed to supplement the element dilution zone caused by diffusion. The diffusion is stronger at high temperature. From the diffusion range, the diffusion range of Cr is 0.162 mm which is larger than that of Fe, and the diffusion range of Fe is 0.161 mm which is larger than that of Mo with the diffusion range 0.12 mm, which also shows that the diffusion rate of multi-element decreases in turn, and Mo is the slowest. The driving force of element diffusion comes from the concentration gradient. 

**Figure 13:**
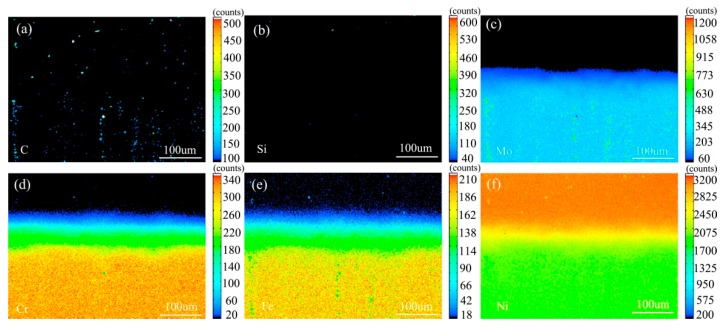
Composition distribution map of the diffusion couple sample of 0Si alloy and pure nickel after 1000 °C/200 h heat treatment: (**a**) C, (**b**) Si, (**c**) Mo, (**d**) Cr, (**e**) Fe, and (**f**) Ni.

Figure 13 shows the element distribution map of the diffusion couple sample of 0Si alloy and pure nickel after 1000 °C/200 h heat treatment by EPMA. It can be seen from the element distribution map of C that carbon elements are distributed in alloy and pure nickel, and the diffusion distance is far, and the diffusion speed is the fastest. Si distribution map shows that there is no Si in the alloy and pure nickel. Cr, Fe, and Mo are also diffused into pure nickel. It can be seen clear from the comparison of the distribution map of these elements, the diffusion range of Cr and Fe is obvious, while the diffusion range of Mo is the smallest. In addition, from the diffusion range of Cr and Fe, after elements near the interface diffuse to pure nickel, the elements in the matrix can quickly supplement to the dilution layer, so there are diffusion layers with different concentrations (reflected by color). However, when Mo diffuses into pure nickel at the interface, the diffusion ability of elements in the matrix to Mo dilution layer is limited, so there is no diffusion character like Cr and Fe. Compared with the EPMA map at low temperature, the diffusion range is obvious. The diffusion distance of iron is the largest.

(b) 1000 °C/200 h diffusion of pure nickel-2Si alloy

**Figure 14:**
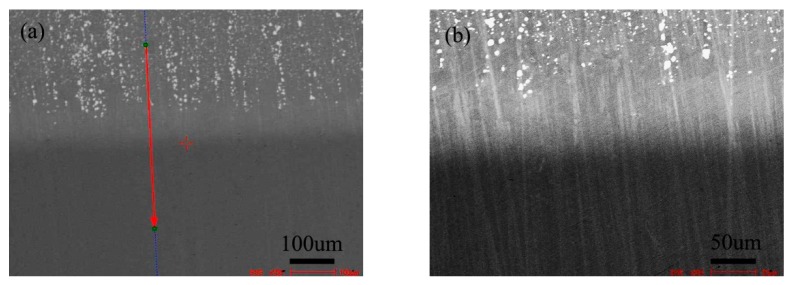
Microstructure of diffusion couple sample after 1000 °C/200 h heat treatment: (**a**) Composition line scan position of the sample and the upper side is 2Si alloy and Lower side is pure nickel, (**b**) the enlarged view of map a.

Figure 14 shows the microstructure of the diffusion couple composed of 2Si alloy and pure nickel after diffusion at 1000 °C/200 h. The upper side of the figure is the microstructure of silicon containing alloy and the lower side is the microstructure of pure nickel. The first map is the location map of line scanning, and the second is the enlarged map of the first map.

**Figure 15:**
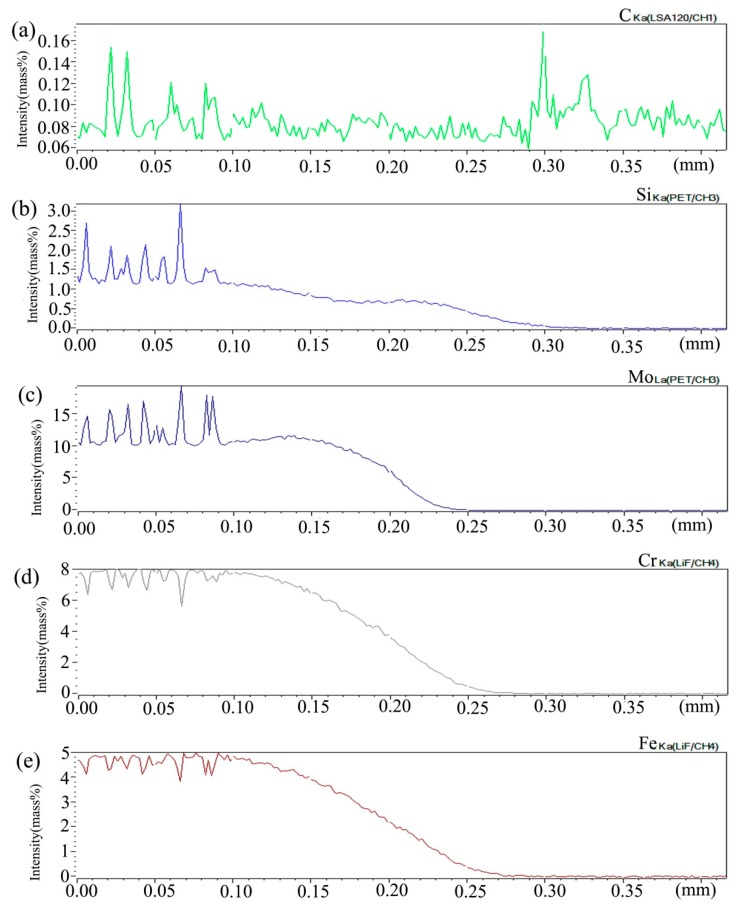
Composition distribution curve of the diffusion couple sample of 2Si alloy and pure nickel after 1000 °C/200 h heat treatment: (**a**) C, (**b**) Si, (**c**) Mo, (**d**) Cr, and (**e**) Fe. (The horizontal coordinate of the figures is the scanning distance and the vertical coordinate is element signal intensity, the zero position is the starting point of scanning).

Figure 15 shows the distribution curve of Mo, Cr, Fe, C, and Si in the diffusion couple after diffusion. The bonding position of diffusion couple interface is at 0.209 mm position, the left side of the figure is 2Si alloy and the right side is pure nickel. From the diffusion curve of C element, it can be seen that the curve on both sides is almost the same height, there is no significant concentration gradient change, and the diffusion speed is very fast. In the measurement range, the diffusion distance is far. From the diffusion curve of Si, it can be seen that Si is still the element with a diffusion speed next to that of carbon, much higher than that of Cr, Fe, and Mo. The diffusion curve of other elements shows that compared with the low temperature diffusion, the diffusion speed of all elements is faster and the diffusion distance is longer when the temperature is increased and the diffusion time is the same. There is still a significant concentration gradient, but the trend of the curve on the left and right sides of the interface is showing clearly that under the high-temperature diffusion condition, compared with the low-temperature diffusion, the highest component curve of each element on one side of the alloy moves towards the interface position, which shows that after the element near the interface diffuses to pure nickel at high temperature, the element in the matrix can diffuse to the interface at a faster speed to supplement the element The dilution caused by diffusion is stronger at high temperature. From the diffusion range, the diffusion range of Si is 0.22 mm, Cr is 0.18 mm, Fe is 0.18 mm, and Mo is 0.12 mm, which also shows that the diffusion rate of multi-element decreases in turn, and Mo is the slowest. The driving force of element diffusion comes from the concentration gradient.

**Figure 16:**
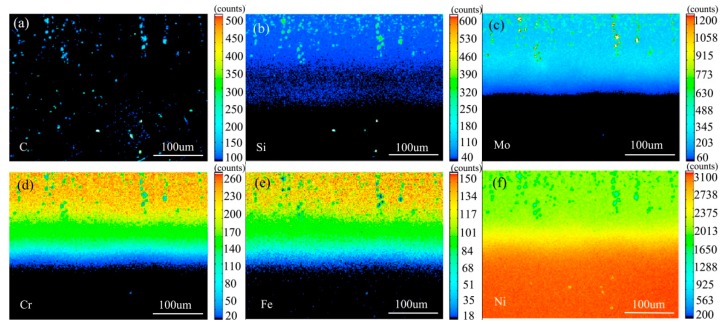
Composition distribution map of the diffusion couple sample of 2Si alloy and pure nickel after 1000 °C/200 h heat treatment: (**a**) C, (**b**) Si, (**c**) Mo, (**d**) Cr, (**e**) Fe, and (**f**) Ni.

Figure 16 shows the element distribution map of the diffusion couple sample of 2Si alloy and pure nickel after 1000 °C/200 h heat treatment by EPMA. It can be seen from the element distribution map of C that carbon elements are distributed in alloy and pure nickel, and the diffusion distance is far, and the diffusion speed is the fastest. Si distribution map shows that Si in the alloy diffuses into pure nickel at a faster speed. Cr, Fe, and Mo also diffused into pure nickel. Compared with the distribution map of the elements, it can be seen clearly that the diffusion range of Cr and Fe is obvious, while the diffusion range of Mo is the smallest. In addition, from the diffusion range of Cr and Fe, after elements near the interface diffuse to pure nickel, the elements in the matrix can quickly supplement to the dilution layer, so there are diffusion layers with different concentrations (reflected by color). However, when Mo diffuses into pure nickel at the interface, the diffusion ability of elements in the matrix to Mo dilution layer is limited, so there is no diffusion character like Cr and Fe. Compared with the EPMA diagram at low temperature, the diffusion range is obviously enlarged. The diffusion distance of iron is the largest. The analysis and comparison of Mo map show that there is no secondary phase precipitated along the grain boundary, mainly at high temperature of 1000 °C.

### 3.2. Interaction of Carbon and Silicon on Microstructure and Stress Rupture Properties

#### 3.2.1. Microstructure and Properties of the Alloy with Same Silicon Content and Different Carbon Content

**Figure 17:**
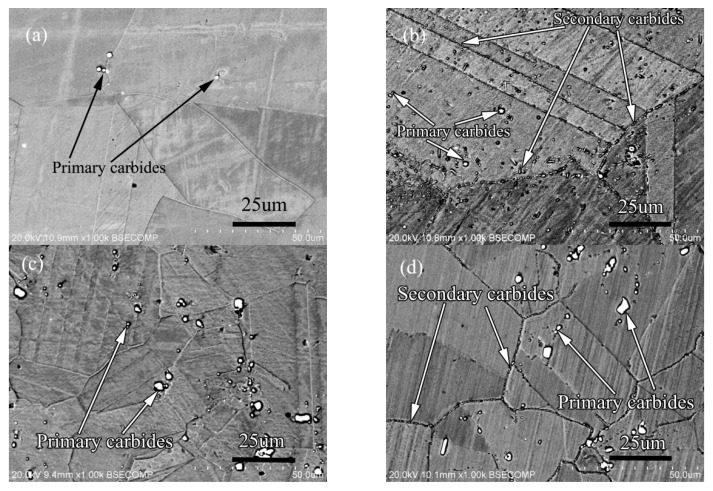
Microstructure of alloy: (**a**) 0.011C alloy solid solution state, (**b**) 0.01C alloy aging state, (**c**) 0.045C alloy solid solution state, (**d**) 0.045C alloy aging state.

In order to further analyze the influence of Si on the secondary phase precipitation behavior of the alloy, and to study the influence of the interaction between carbon and silicon on the structure and properties of the alloy, the alloy 3 and alloy 4 were prepared. The carbon content of alloy 3 is 0.45 wt.%, and that of alloy 4 is 0.11 wt.%. The other alloy components are the same, and the content of silicon element is same too. The solid solution state and aging state microstructure of the alloy are shown in Figure 17.

The solid solution structure of the two alloys is shown in Figure 17a,c. Compared with the solid solution structure of the two alloys, it can be seen that the distribution of primary carbides are similar, and both are in the grain boundary and in the grains at the same time, but the quantity and size are very different. The primary carbides of the low-carbon alloy are significantly less than those of the high-carbon alloy, and the size of the primary carbides in the low-carbon alloy is also smaller than that in the high-carbon alloy. After aging treatment, the precipitation of secondary carbide in low carbon alloy is different from that in high carbon alloy as shown in Figure 17b,d. From the amount of secondary precipitation, the amount of secondary precipitation in low carbon alloy is significantly higher than that in high carbon alloy. From the point of view of precipitation location, the secondary phase of low-carbon alloy precipitates in the grain boundary, twin boundary and grains, but the secondary phase precipitates only in the grain boundary of high-carbon alloy. The size of the secondary phase of low carbon alloy is slightly larger than that of high carbon alloy. According to the results of phase identification, the primary carbides of low carbon alloy and high carbon alloy are M_6_C type carbides, while the secondary precipitation carbides of the two alloys are all M_12_C type carbides.

**Figure 18:**
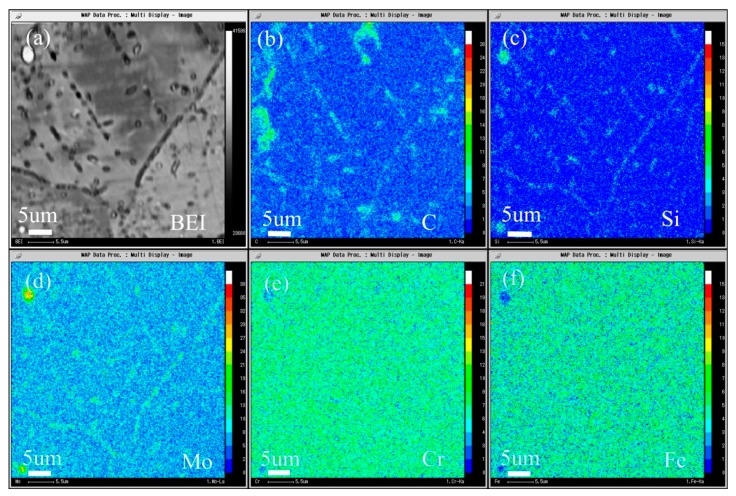
Microstructure of the 0.011C specimen aged at 900 °C for 2 h: (**a**) BEI image and the area distribution of (**b**) C, (**c**) Si, (**d**) Mo, (**e**) Cr, and (**f**) Fe by EPMA.

The composition distribution map of low carbon alloy with carbon content of 0.011 wt.% after solution and aging treatment scanned by EPMA is shown in Figure 18, and Figure 18a is the backscatter diagram of the alloy. It can be seen from the map that there are secondary phase precipitation in the grain boundary, twin boundary, and grains. It can be seen from Figure 18b–d that both primary carbide and secondary carbide contain C and Si, and Mo is also enriched. It can be seen from Figure 18e,f that the primary carbide and the secondary precipitated carbide are slightly different, the primary carbide Cr and Fe are depleted, but the secondary precipitated phase is not obvious.

**Figure 19:**
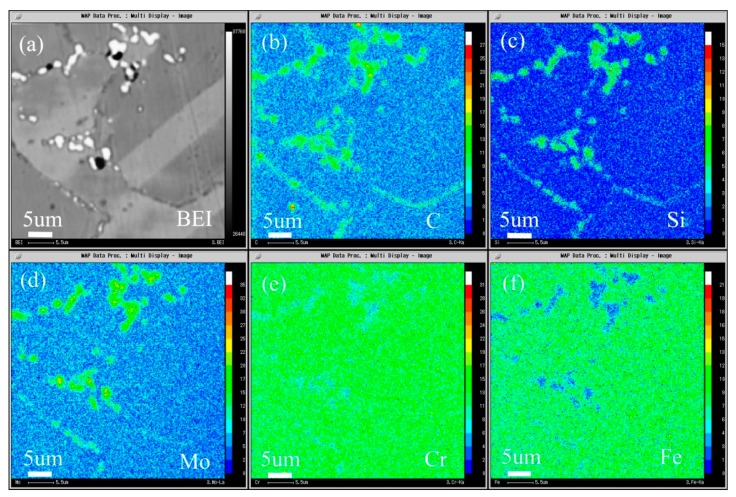
Microstructure of the 0.045C specimen aged at 900 °C for 2 h: (**a**) BEI image and the area distribution of (**b**) C, (**c**) Si, (**d**) Mo, (**e**) Cr, and (**f**) Fe by EPMA.

The composition distribution map of low carbon alloy with 0.045 wt.% carbon content after solution and aging treatment scanned by EPMA is shown in Figure 19, and Figure 19a is the backscattering map of the alloy. It can be seen from the map that, unlike low carbon alloy, the secondary phase of high carbon alloy only precipitates at the grain boundary. It can be seen from Figure 19b–d that both primary carbide and secondary carbide contain C and Si, and Mo is also enriched. It can be seen from Figure 19e,f that the primary carbide and secondary precipitated carbide are slightly different, the primary carbide Cr and Fe are depleted, while the secondary precipitated phase is not obvious, but it is more significantly depleted than low carbon alloy.

The stress rupture life data of two alloy samples in solution and aging state are shown in Table 2. It can be seen from the experimental results that the stress rupture life of low-carbon alloy in solution state is less than half of that of high-carbon alloy, which is significantly lower than that of high-carbon alloy. After aging treatment, the stress rupture life of the two alloys is obviously increased at the same time. However, the stress rupture life of low carbon alloy increased by 286.4% from 22 h to 85 h, while that of high carbon alloy increased by 61.7 h from 47 h to 76 h. At the same time, the aging life of low carbon alloy is 85 h, which is also longer than that of high carbon alloy.

#### 3.2.2. Microstructure of the Alloy with Different Silicon Content and Same Carbon Content

**Figure 20:**
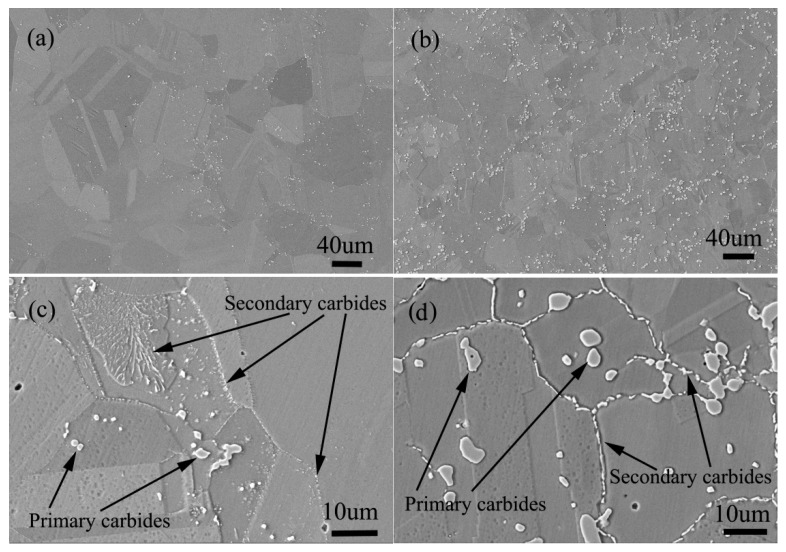
Microstructure of solid solution state alloy: (**a**) 0Si alloy, (**b**) 2Si alloy; Microstructure of aging state alloy: (**c**) 0Si alloy, (**d**) 2Si alloy.

The solid solution structure and aging structure of the two alloys with the same carbon content of 0.045 (wt.%) and silicon content of 0 and 0.45 (wt.%) respectively are shown in Figure 20.

It can be seen from Figure 20a,b that the amount of primary carbides in silicon free alloy is significantly less than that in silicon containing alloy, and the size of carbides in silicon free alloy is also significantly smaller than that in silicon containing alloy. The distribution of primary carbides in the two alloys is the same, both in the grain boundary and in the grains. After aging treatment, secondary carbides are precipitated along the grain boundary as shown in Figure 20c,d, but compared with the secondary precipitates of the silicon containing alloy, the secondary precipitates of the alloy without silicon are less in amount and smaller in size. The most important thing is that the carbide type of secondary precipitates is different, the type of silicon free alloy is M_6_C, and the type of silicon containing alloy is M_12_C. According to the author’s early experimental research [1,2], because M_12_C has a coherent orientation relationship with the matrix, the alloy containing M_12_C carbide has better stress rupture properties.

## 4. Discussion

### 4.1. Characteristics of Carbides M_6_C and M_12_C

The sketch maps of the carbides M_6_C and M_12_C have been reported [1], there are significant differences in the structure of the carbides, the distribution of space group and metal atoms are the same. The number, distribution and lattice constant of carbon atoms are different. M_12_C is 10.86 Å, which is smaller than that of M_6_C which is 11.08 Å [34]. At the same time, the formation of two kinds of carbides all requires more than two kinds of metal elements. The formation free energies of M_6_C and M_12_C are −77.2 kJ/mol and −130kJ/mol respectively [35], so M_12_C is easier to form than M_6_C. M_12_C will be formed preferentially in the relatively low carbon and high carbide forming element enrichment area where M_6_C cannot be formed. Compared with structure characteristics of the two carbides, the formation of M_12_C needs more metal elements.

According to the type of carbide precipitated in aging process, silicon atom occupies the position of metal atom. This is because if it occupies the position of carbon atom, it does not conform to the formation characteristics of carbide, so it is equivalent to the increase of carbon atom. When aging, it is more appropriate to precipitate M_6_C type carbide than M_12_C type carbide. It is the relative depletion of carbon atoms that makes M_12_C precipitate more easily than M_6_C.

From the analysis of the precipitated carbide composition, the higher the silicon content in the alloy, the higher the silicon content in the carbide, which shows that silicon does not occupy the original carbon atom position, otherwise it will not change the proportion. The more silicon is occupied, the more alternative metal atoms are occupied.

From the analysis of the amount of precipitated carbides, it can be confirmed that under aging temperature, silicon can promote the precipitation of secondary phase. The reason why there are many secondary phases in low-carbon alloy is that the primary phase of high-carbon alloy precipitates more carbide and consumes more silicon because of high carbon content, which leads to relatively high silicon content in low-carbon alloy during aging, so more secondary phases are precipitated. It also shows that silicon should occupy metal atom position in carbide structure.

### 4.2. Effect of Silicon on Element Diffusion

According to the experimental results, the diffusion process with the same carbon content and different silicon content did not show the upward diffusion of carbon. When the silicon content is different and other elements are the same, silicon has a significant effect on the diffusion of other elements. Each element diffuses also according to the concentration difference. When the diffusion couple composed of alloy and pure nickel diffuses, silicon promotes the diffusion of other elements—such as C, Cr, Mo, and Fe—and improves the diffusion speed of these alloy elements. At the same time, each element diffuses in the direction of concentration gradient. Silicon diffuses faster than other metals. In the process of alloy element diffusion, molybdenum element diffusion is the slowest because of its larger atomic size. For each diffusion couple, the diffusion rate of various elements is faster at 1000 °C than at 800 °C. High temperature accelerated the diffusion rate of various elements.

According to the experimental results, silicon has a much higher diffusion rate than other metal elements. Silicon has a certain effect on the diffusion of elements in the alloy. At 800 °C and 1000 °C, silicon improves the diffusion rate of metal elements in the alloy. The increase of element diffusion speed is helpful to the enrichment of metal elements in carbide forming area and the precipitation of carbide.

More metal atoms are needed for M_12_C than for M_6_C with the same number of carbon atoms. Therefore, it can be inferred that the change of secondary phase precipitation type by silicon is due to the increase of diffusion speed of metal elements and the relative enrichment of metal elements. Therefore, compared with silicon-free alloy, M_12_C is more likely to be formed than M_6_C.

### 4.3. Effect of Silicon on Primary Phase of as Cast Alloy

According to the experimental results, under the same carbon content and other alloy elements, the primary carbides of silicon free alloy and silicon alloy are M_6_C type carbides, that is to say, silicon element has no effect on the type of primary carbides. However, the amount of primary carbides precipitated from the silicon containing alloy is significantly larger than that from the alloy without silicon, and its size of the silicon containing alloy is also significantly larger than that from the silicon free alloy, which indicates that silicon can promote the precipitation of primary carbides (comparison between 0Si alloy and 2Si alloy). However, there is no significant effect on the distribution of primary carbides. The primary carbides of the two alloys are mainly distributed in the grain boundary and grains. The type of carbides are all also M_6_C for these two alloys. It can be seen that Si has no effect on the type of primary carbide. It is believed that the most important C element formed by alloy elements and carbides can diffuse to the grain boundary in time under high temperature conditions, and meet the formation conditions of M_6_C carbides. Combined with the research results of Million B et al. [11], the bonding force of Si has been completely destroyed at high temperature, which will not affect the binding of alloy elements. Therefore, the type of primary carbides formed in the solidification process of the alloy has not been affected by Si. However, because Si promotes the diffusion of alloy elements and Si itself participates in the formation of carbides, the primary carbides of high Si alloy precipitate more when the carbon content is the same.

### 4.4. Effect of Silicon on the Type of Secondary Phase of Alloy

According to the experimental results, when the carbon content and other alloy elements are the same, the addition of silicon has a significant effect on the precipitation behavior of the secondary precipitation phase. First of all, it is also the most important point that silicon affects the type of carbide secondary precipitation in the alloy. The secondary precipitation of the alloy with silicon is M_12_C, and the secondary precipitation of the alloy without silicon is M_6_C. The second point is silicon has influence on the amount and size of the secondary phase. The amount of secondary phase precipitation in silicon free alloy is less than that in silicon containing alloy, and the size is significantly smaller than that in silicon containing alloy. The influence mechanism of silicon on the secondary phase precipitation of alloy can be revealed by combining the effect of silicon on the element diffusion. The addition of silicon improves the diffusion speed of alloy elements. At the grain boundary, a large amount of carbides forming metal elements are enriched. Compared with the silicon free alloy, the silicon containing alloy has more carbide forming elements enriched at the grain boundary, and a large amount of Si enriched at the grain boundary at the same time for the silicon containing alloy. The element Si can occupy the metal atom position, and can replace some metals to form carbides, which results in the relative shortage of element C of the silicon containing alloy. Because M_12_C has lower formation free energy than M_6_C, it is more beneficial to formation, because of the enrichment of metal elements, which contributes to the formation of M_12_C.

In essence, the influence of Si on the precipitation type, quantity and size of the secondary phase is caused by the influence of Si on the diffusion speed of alloy elements. Si increases the diffusion rate of metal elements, resulting in the enrichment of metal elements at the grain boundary. Si itself has a faster diffusion rate than metal elements at the same time, Si occupies the metal atom position when carbides are formed, replacing some metal atoms. This results in the relative deficiency of C in the grain boundary. Because M_12_C has lower formation free energy than M_6_C, it is easier to form. All these factors lead to the formation of M_12_C type carbide.

Because Si can promote the diffusion of elements, which can accelerate the formation and growth of carbides, the amount and size of secondary phase precipitation in Si containing alloys are larger. With the same carbon content and different silicon content, the primary carbide is less when silicon content is low. After aging, there is still such a rule. With the same carbon content, the secondary precipitates of silicon containing alloy are more. It shows that silicon can promote the precipitation of carbides. Silicon promotes the precipitation of carbides in the process of solidification at high temperature and aging at low temperature.

### 4.5. Effect of Interaction Between Carbon and Silicon on Primary and Secondary Phases of Alloy

Silicon content (0.45 wt.%) other metal content is the same, carbon content is 0.01 and 0.045 respectively, compared with primary carbides of these alloys, it is found that the amount and size of carbides precipitated in low carbon alloy are smaller. This shows that the precipitation of primary carbide is not only affected by silicon, but also by carbon.

Among alloys with same silicon and metal element contents, the alloy which has more carbon sees easier carbide precipitation, and the carbides be of greater quantity and larger size. However, after aging treatment, the precipitation behavior of the secondary precipitates of the two alloys has changed differently. The amount and size of secondary phase precipitation of low carbon alloy are larger than that of high carbon alloy, and the precipitation location of the low carbon alloy is more than that of the high carbon alloy. The secondary carbide of the low carbon alloy precipitates in the grain boundary, twin boundary and grains. However, there are only secondary precipitates in the grain boundary for the high carbon alloy, and the amount and the size of the carbides are all smaller than that of the low carbon alloy.

The reason is that when the silicon content and alloy elements are the same, the carbon content determines the amount of primary carbides. The more carbon elements are, the more primary carbides will be precipitated, and the more primary carbides will be precipitated, the more silicon elements will be consumed at the same time. As a result, the low-carbon alloy becomes a relatively high silicon alloy during aging compared with the high carbon alloy. The high carbon alloy becomes a relative low silicon alloy, and the carbon content of the alloy is also significantly reduced because of the massive precipitation of the primary carbides. In the aging process, the silicon element has a greater effect on the secondary phase precipitation, because the silicon element consumption of high carbon alloy is more, the remaining is less. In contrast, low-carbon alloy has more silicon content, which promotes the precipitation of the secondary phase more obviously than the high carbon alloy. Therefore, in the aging process, the low-carbon alloy precipitates more secondary phase precipitation than the high carbon alloy. However, the carbon content does not affect the precipitation type of carbides. The primary carbides of the two alloys are all M_6_C type carbides, while the secondary precipitation carbides are all M_12_C type carbides.

### 4.6. Effect Essence of Silicon and Carbon Interaction on Properties of the Alloy

According to the comparison of the stress rupture life of alloy 2 and alloy 3, under the same silicon content, the stress rupture life of solution state low carbon alloy is lower than that of high carbon alloy. This is because the amount of primary carbides in the solid solution microstructure of low-carbon alloy is significantly less than that of high-carbon alloy. Under the condition of 650 °C/325 MPa stress rupture test, carbides can significantly improve the stress rupture life of the alloy, so the stress rupture life of high-carbon alloy with more M_6_C carbides is better than that of the low carbon alloy. After aging treatment, the stress rupture lives of the two alloys are all longer than that of their solution state. However, the increasing range of stress rupture life of the two aging state alloys is very different, and the increasing range of low-carbon alloy is the largest, and the stress rupture life of aging state low-carbon alloy is longer than that of high-carbon alloy. The reason why the stress rupture life of the two aging state alloys increases is that the fine and dispersed M_12_C secondary carbides are precipitated along the grain boundary after aging heat treatment and the grain boundary is effectively strengthened, so the stress rupture life of the two aging state alloys is longer than that of their solid solution alloys. The increasing range difference is that due to the promotion of silicon element on carbide precipitation, for the low carbon alloy, there are fine and dispersed secondary strengthening phases in the grain boundary, twin boundary, and grains of low carbon alloy, with a greater amount of secondary carbides, more obvious strengthening effect of alloy, and greatly improved properties of the alloy. Therefore, the life of low carbon alloy after aging is longer than that of high carbon alloy. In essence, the difference between the two alloys is the result of the joint action of primary carbide and secondary precipitation carbide.

For the alloys with same carbon content, the stress rupture life of silicon-containing alloy is higher than that of silicon-free alloy both in the solution state and in the aging state [1,2]. It is caused by the difference of alloy microstructure. In the solution state, the primary carbide in silicon-free alloy is significantly less than that of silicon-containing alloy, so the strengthening effect of primary carbide of silicon-containing alloy is more than that of silicon free alloy. Therefore, the stress rupture life is longer. After aging treatment, the stress rupture life of the two alloys are all increased, which is due to the secondary phase precipitation at the grain boundary after aging, strengthening the grain boundary and improving the stress rupture strength. However, the increase range is different, because the secondary precipitate of silicon free alloy is M_6_C, while the secondary precipitate of silicon containing alloy is M_12_C. The M_12_C has a coherent orientation relationship with the matrix, so the strengthening effect is better.

Alloy 3, at different states, has a different stress rupture life. The stress rupture life at the aging state is much higher than that of the alloy at the solution state. That is because after aging treatment, there are many secondary carbides precipitate along the grain boundary, the secondary carbides improved the stress rupture life of the alloy apparently. 

Two groups of experimental results with different carbon content and different silicon content are all caused by the different primary and secondary carbide precipitation behaviors in the microstructure of the alloy, which is caused by the joint action of carbon and silicon elements on the carbide precipitation behavior of the alloy. The effect of silicon on the precipitation behavior of alloy is essentially the effect on the diffusion of elements in the alloy.

## 5. Conclusions

1. In the Ni-Mo-Cr-Fe alloy matrix, silicon has a faster diffusion rate than Cr, Fe, and Mo, and at the temperatures ranging from 800 °C to 1000 °C, silicon can promote the diffusion of Cr, Fe, and Mo. The diffusion process is from high concentration range to low concentration range, and there is no upslope diffusion of carbon element. The higher the temperature is, the stronger the diffusion ability of alloy elements is.
2. Silicon can promote the precipitation of alloy carbide, whether it is the primary carbide formed by casting or the secondary carbide precipitated by aging. With the same carbon content, the higher the silicon content, the more primary carbides will form. With the same silicon content, the higher carbon content, the more primary carbides will form. Silicon can also promote the precipitation of the secondary phase in the alloy. With the same carbon content, more silicon content means more secondary phase.
3. The effect of silicon on the type of precipitates is related to temperature, which only affects the amount of primary carbides, while silicon has no effect on the type of primary carbides formed in the casting process.
4. The secondary precipitation behavior (type, quantity, and distribution of precipitates) is the result of the joint action of carbon and silicon elements in the alloy, and it is also a complex process related to the thermal history of the alloy. For the alloy with 0.045C (wt.%), the secondary phase precipitates only at the grain boundary, while for the alloy with 0.011C (wt.%), the secondary phase precipitates not only at the grain boundary, but also in the intragranular and twin boundaries. However, the secondary precipitates of the two Si containing alloys with different carbon contents are all M_12_C type carbides.
5. Under low carbon condition, the formation free energy of M_6_C is higher than that of M_12_C, and M_12_C is easier to form. Silicon is more likely to reduce the carbon content of the alloy. Because silicon can promote the formation of carbides and consume more carbon elements, when the carbon content of the two alloys is the same, the alloy containing silicon contains more primary carbides and consumes more carbon in the solid solution state. During aging, the carbon elements diffused to the grain boundary are relatively reduced, which is not easy to form M_6_C, but easier to form M_12_C.
6. M_12_C has a higher strengthening effect on the alloy than M_6_C, which can significantly improve the stress rupture strength of the alloy than M_6_C.

## Figures and Tables

**Table 1:** Chemical composition of the experimental alloy (wt.%)

Alloy/Element	Si	C	Mo	Cr	Fe	Ni
Alloy 1	0	0.045	15.8	6.99	4.11	Bal.
Alloy 2	0.09	0.045	15.6	6.97	4.08	Bal.
Alloy 3	0.45	0.045	16.1	7.02	4.32	Bal.
Alloy 4	0.45	0.011	15.8	6.98	4.23	Bal.
Alloy 5	1.96	0.045	16.0	6.96	4.22	Bal.

Attention: (1) Alloy 2 and alloy 3 are used for making diffusion samples together; (2) alloy 1 and alloy 5 are used to make diffusion samples with pure nickel alloy; (3) the alloy 3 and alloy 4 are used for stress rupture testing.

**Table 2:** Stress rupture life of the alloys with different C content

Alloy	C Content	Heat Treatment	Test Condition	Stress Rupture Life
Alloy 2	0.11	1180 °C/1 h	650 °C/325 MPa	22 h
Alloy 3	0.45	1180 °C/1 h	650 °C/325 MPa	47 h
Alloy 2	0.11	1180 °C/1 h + 900 °C/2 h	650 °C/325 MPa	85 h
Alloy 3	0.45	1180 °C/1 h + 900 °C/2 h	650 °C/325 MPa	76 h
